# Efficacy of Silver Nanoparticles-Loaded Bone Cement against an MRSA Induced-Osteomyelitis in a Rat Model

**DOI:** 10.3390/medicina59040811

**Published:** 2023-04-21

**Authors:** Young Suk Choi, Young Hwan Kim, Hye Min An, Sung Kyoung Bae, Young Koo Lee

**Affiliations:** 1 Department of Biology, Soonchunhyang University, 22, Soonchunhyang-ro, Asan-si 31538, Chungcheoungnam-do, Republic of Korea; imysuk@hanmail.net; 2 Department of Orthopedic Surgery, Soonchunhyang University Bucheon Hospital, 170, Jomaru-ro, Wonmi-gu, Bucheon-si 14584, Gyeonggi-do, Republic of Korea; remedios@schmc.ac.kr (Y.H.K.); ahm112597@naver.com (H.M.A.); qotjdrud00@naver.com (S.K.B.); 3 Department of Medical Sciences, Soonchunhyang University, 22, Soonchunhyang-ro, Asan-si 31538, Chungcheoungnam-do, Republic of Korea

**Keywords:** bone cement, AgNP, osteomyelitis, MRSA, bone regeneration

## Abstract

*Background and Objectives:* The purpose of this study was to assess the cytotoxicity and antibacterial effects of AgNP-impregnated Tetracalcium phosphate-dicalcium phosphate dihydrate (TTCP-DCPD). *Materials and Methods:* Using in vitro experiments, the cytotoxicity of AgNP-impregnated TTCP-DCPD against fibroblasts and osteocytes was assessed in terms of cell viability by water-soluble tetrazolium salt assay. To assess antibacterial effects, a disc diffusion test was used; osteomyelitis was induced first in vivo, by injection of methicillin-resistant *Staphylococcus aureus* into the tibia of rats. AgNP-impregnated TTCP-DCPD bone cement was then applied at various silver concentrations for 3 or 12 weeks. Antibacterial effects were assessed by culturing and reverse transcription-polymerase chain reaction (RT-PCR). For histological observation, the bone tissues were stained using hematoxylin and eosin. *Results:* Cell viability was decreased by the impregnated bone cement but did not differ according to AgNP concentration. The diameter of the growth-inhibited zone of MRSA was between 4.1 and 13.3 mm on the disks treated with AgNP, indicating antimicrobial effects. In vivo, the numbers of bacterial colonies were reduced in the 12-week treatment groups compared to the 3-week treatment groups. The groups treated with a higher (10×) dose of AgNP (G2–G5) showed a tendency of lower bacterial colony counts compared to the group without AgNP (G1). The PCR analysis results showed a tendency of decreased bacterial gene expression in the AgNP-impregnated TTCP-DCPD groups (G2–G5) compared to the group without AgNP (G1) at 3 and 12 weeks. In the H&E staining, the degree of inflammation and necrosis of the AgNP-impregnated TTCP-DCPD groups (G2–G5) showed a tendency to be lower at 3 and 12 weeks compared to the control group. Our results suggest that AgNP-impregnated TTCP-DCPD cement has antimicrobial effects. *Conclusions:* This study indicates that AgNP-impregnated TTCP-DCPD bone cement could be considered to treat osteomyelitis.

## 1. Background

Hematogenous osteomyelitis accounts for approximately 20% of all osteomyelitis cases worldwide, the majority of which are pediatric [1]. However, the prevalence of this condition is declining due to improved social and economic conditions, as well as advances in medical technology [2]. In comparison to hematogenous osteomyelitis, cases of contiguous osteomyelitis increased due to a rise in the number of traffic accidents and arthroplasty procedures. Contiguous osteomyelitis, which accounts for approximately 80% of all osteomyelitis cases, is broadly classified as acute, subacute or chronic, but it can also be classified in other ways; for example, in accordance with complications or disease site [2]. Acute osteomyelitis is characterized by an inflammatory response, whereas chronic osteomyelitis results in bone necrosis [3]. Methicillin-resistant *Staphylococcus aureus* (MRSA) is among the major pathogens detected in osteomyelitis [4]. Bacterial infection induces an inflammatory response in bones and surrounding tissues, resulting in bone loss, dysfunction and osteoblast apoptosis [5]. Osteomyelitis is frequently treated surgically or with antibiotic therapy. In the surgical method, the pathogen is removed, along with the infected tissue, and necrotic tissues are eliminated by debridement [6]. Any remaining infection is treated by antibiotic therapy. Antibiotic-impregnated bone cements are effective; however, during the treatment of osteomyelitis, long-term antibiotic therapy (oral or intravenous administration) can induce severe adverse events [7]. Injected or orally administered antibiotics can circulate systemically, affecting not only the lesion site but also normal tissues, causing various side effects. Antibiotic-impregnated bone cement can act directly at the lesion site, thereby eliminating the side-effects of general antibiotics administration [8]; an additional advantage is the delivery of high-dose antibiotics to lesion sites. Site-specific drug delivery systems, utilizing non-biodegradable materials such as polymethylmethacrylate or biodegradable and osteoactive materials such as calcium orthophosphates bone cements, were demonstrated to be effective and promising alternatives for the management of osteomyelitis [9]. Calcium orthophosphate cement formulations are typically composed of an equimolar mixture of tetracalcium phosphate (TTCP) and dicalcium phosphate (DCPA or DCPD) [10]. Silver nanoparticles with antimicrobial effects are widely used in various applications such as bone cement, biomaterials, implant coatings, and joint restoration materials, indicating potential applications in the field of bone regeneration [11,12,13]. Materials such as calcium phosphate and tetracalcium phosphate-dicalcium phosphate dihydrate (TTCP-DCPD) bone cements can repair bone defects due to their osteoconduction and osseointegration properties [6]. Due to their high biocompatibility, these materials can also regenerate bones and release drugs effectively (as a carrier or scaffold) [14]. This study examined the effects of silver nanoparticle (AgNP)-impregnated bone cement on osteomyelitis.

## 2. Methods

### 2.1. Fibroblast Isolation from Skin Tissue

Skin tissue was obtained from 8-week-old Sprague Dawley rats (Orient Bio Inc., Gyeonggi-do, Republic of Korea). The rats were anesthetized using a 4:1 mixture of Zoletil (Virbac Animal Health, Carros, France) and Rumpun (Bayer Korea, Seoul, Republic of Korea). Skin tissue biopsy was then obtained after trimming their abdominal fat and washing the skin tissues with phosphate-buffered saline (PBS) and mincing into 0.5–1-mm^3^ pieces. The pieces were placed in six-well plates containing Dulbecco’s modified Eagle’s medium/Ham’s nutrient F-12 mixture (DME/F12) supplemented with 20% fetal bovine serum (FBS) and 50 µg/mL gentamicin; the plates were incubated at 37 °C in a 5% CO_2_ atmosphere. After 24 h, the tissues were removed for isolation of fibroblasts. Primary skin fibroblasts were maintained for 2–3 weeks in DME/F12 supplemented with 10% FBS and 50 µg/mL gentamycin, and were incubated at 37 °C in a 5% CO_2_ atmosphere [15].

### 2.2. Osteocytes Isolation from Calvaria

To isolate osteocytes, 1–4-day-old Sprague Dawley rats (Orient Bio Inc.) were euthanized. Their cortical bones were removed and washed with 70% ethanol approximately 15 times; they were then washed with PBS three times, for 5 min each. Pieces of bone were digested by collagenase type 1 (Sigma-Aldrich, St. Louis, MO, USA) dissolved in PBS (with calcium) for 1 h at 37 °C. After centrifugation at 200× *g* for 5 min, the obtained pellets were placed in a culture plate containing minimum essential medium (MEM) alpha medium supplemented with 20% FBS and 50 µg/mL gentamycin, followed by incubation at 37 °C in a 5% CO_2_ atmosphere. The remaining pieces of bone were washed with PBS and incubated with 5 mM ethylenediaminetetraacetic acid in PBS and 0.1% bovine serum albumin (BSA) for 20 min, at 37 °C in a 5% CO_2_ atmosphere. The supernatants were then collected and centrifuged at 200× *g* for 5 min, and the pellets transferred to plates as described above. After 3 weeks, osteocytes were maintained in MEM alpha medium supplemented with 10% FBS 50 µg/mL gentamycin, at 37 °C in a 5% CO_2_ atmosphere [16].

### 2.3. Cell Characterization

Immunostaining was used to characterize the isolated primary fibroblasts and osteocytes. Briefly, 5 × 10^4^ cells were seeded in a 24-well plate, followed by fixation using 4% paraformaldehyde for 10 min. For permeabilization, samples were incubated with PBS in 0.25% Triton X-100 for 10 min at 25 °C. After washing with PBS, samples were incubated with 1% BSA in PBST for 30 min at 25 °C. The primary antibody used to stain fibroblasts was goat anti-rabbit IgG H&L (Abcam, Cambridge, UK). FITC-conjugated anti-podoplanin polyclonal antibody (Bioss Inc., Woburn, MA, USA) was used for osteocytes. Fibroblasts and osteocytes were treated with the primary antibodies overnight at 4 °C. The secondary antibodies used were rat monoclonal IgG2a (Santa Cruz Biotechnology, Inc., Dallas, TX, USA) for fibroblasts and goat anti-rabbit IgG-HRP (Santa Cruz Biotechnology) for osteocytes, which were incubated with the cells for 45 min. The cells were then washed with PBS; their nuclei were stained using 4′,6-diamidino-2-phenylindole dihydrochloride (DAPI, Sigma-Aldrich) and mounted for microscopy [17].

### 2.4. Cell Characterization

AgNP-containing bone cements (Zimmer Regular, Zimmer Inc., Warsaw, IN, USA) with silver concentrations of 0, 10, 20, 40, 80 or 800 ppm were prepared (10 mm; 1 mm thickness). Approximately 5 × 10^4^ fibroblasts and osteocytes were placed in contact with the AgNP bone cement containing each AgNP concentration. After 1, 4 and 7 days, a water-soluble tetrazolium salt (WST-8; Cell Counting Kit-8^®^, Dojindo Molecular Technologies, Inc., Tokyo, Japan) cell viability assay was carried out to evaluate the cytotoxicity of the AgNP bone cements [18].

### 2.5. Antimicrobial Activity

An antimicrobial disc diffusion test was used to determine the antimicrobial activity of AgNPs (<100 nm particle size, 576832, Sigma-Aldrich, USA) against MRSA (ATCC 43300) by a standardized single disk method [19]. When performing the test, all the samples were cultured on a mannitol salt agar (KP) plate. The surface of the agar was evenly and lightly inoculated using a cotton swab. Prior to inoculation, the swab stick was dipped into bacterial suspension with a turbidity visually equivalent to 0.5 McFarland standards.

Following absorption of the bacterial suspension onto the plates, circular discs containing AgNP solutions diluted in PBS to 0, 10, 20, 40, 80 and 800 ppm were placed on the plate. The plates were incubated for 24 h at 37 °C in a 5% CO_2_ atmosphere, followed by measurement of inhibition zone diameters.

### 2.6. Experimental Animals

In this study, a total of 135 male rats weighing 250–300 g were used. All animals were maintained according to a 12 h light and 12 h dark cycle, at 23 ± 2 °C and with 50 ± 20% humidity. Two rats were maintained in each cage with free access to food and water. All animals were treated in accordance with the guidelines issued by the U.S. National Institutes of Health and Department of Agriculture.

### 2.7. Experimental Osteomyelitis

MRSA was cultured in tryptic soy broth (TSB) for 24 h, diluted 1:100 dilutions with fresh medium and incubated for a further 5 h at 37 °C. A hole approximately 1 mm in diameter was made in the tibiae using a high-speed drill, into which 20 µL of the bacterial suspension (2 × 10^8^ CFU/mL) was injected. The holes were sealed with bone wax and the skin was sutured. After 4 weeks, five rats selected at random were euthanized, their tibiae isolated and osteomyelitis induction assessed. The tibiae were fixed using 4% paraformaldehyde and then sectioned using a microtome (Shandon™ Finessen 325, Thermo Electron Corporation, Waltham, MA, USA) to a thickness of approximately 4 µm. Hematoxylin and eosin (H&E) staining was performed to assess induction of osteomyelitis [20].

### 2.8. Insertion of AgNP-Containing Bone Cement

After 4 weeks of osteomyelitis induction, rats were anesthetized and their legs trimmed. A surgical procedure was carried out to expose their patellar tendons, followed by exposure of their proximal tibial epiphysis; a 1 mm diameter hole was then created using a high-speed drill. To assess the degree of debridement and bone cement filling, holes were also made at the distal tibia. Debridement was performed using an angiocatheter, followed by irrigation of the bone marrow using a saline syringe. AgNP bone cement was then inserted into the bone marrow; the hole was sealed with bone wax and the gaps were sutured. The following AgNP concentrations were used: baseline groups, 0, 10, 20, 40 and 80 ppm; 10× concentration groups, 0, 100, 200, 400 and 800 ppm.

### 2.9. Bacterial Culture

Three or twelve weeks after the insertion of bone cement, three rats in each group were euthanized and their tibiae removed. The tibiae were stored at −80 °C for 24 h, and were then placed into a round-bottom tube containing 2-mL TSB and homogenized. The supernatant was diluted with TSB at a ratio of 1:10^3^–1:10^6^. The bacterial suspensions were smeared onto blood agar plates (20 μL each), which were then incubated at 37 °C in a 5% CO_2_ atmosphere for 24 h. After incubation, the colonies were counted and the CFU/mL calculated [21].

### 2.10. Reverse Transcription Polymerase Chain Reaction (RT-PCR)

Three or twelve weeks after the insertion of bone cement, three rats in each group were euthanized and their tibiae removed. The tibiae were stored at −80 °C for 24 h and then grinded using a pestle and mortar. mRNA was isolated from the ground bone using a Tri-reagent kit (Molecular Research Center, Inc., Cincinnati, OH, USA), chloroform (Sigma-Aldrich) and isopropanol (Sigma-Aldrich); 1 mL Tri-reagent with ground tissue was transferred to an EP-tube and incubated on ice for 5 min. To separate mRNA from tissue, 200-μL chloroform was added and, after incubation, centrifugation at 13,000 rpm was performed for 15 min. The supernatant was then separated into a fresh EP-tube, to which an identical volume of isopropanol was added. The supernatant was incubated on ice for 15 min, followed by centrifugation at 13,000 rpm for 15 min. Finally, the pellet was washed with ethanol and dissolved in 30-μL diethylpyrocarbonate-treated water. To synthesize cDNA, 2 µg of mRNA, 1 µL of random hexamers (Applied Biosystems, Waltham, MA, USA), RNaseOUT™ recombinant ribonuclease inhibitor (Life Technologies, Carlsbad, CA, USA), 1 μL of 10 mM dNTP Mix (Life Technologies) and GoScript™ reverse transcriptase (Promega Corporation, Madison, WI, USA) were used. To detect MRSA, the tuf and *PVL* genes were used as targets, with β-actin as the loading control. The primers for each gene were as follows: β-actin, forward 5′-GATGATATCGCCGCGCTCGTC-3′, and reverse 5′-AGCCAGGT CCAGACGCAGGAT-3′; tuf gene, forward 5′-CAATGCCACAAACTCG-3′, and reverse 5′-GCTTCAGCGTAGTCTA-3′; *PVL* gene, forward 5′-GGCGAGAAAAGCAAGAATTG-3′ and reverse 5′-GTACCTTGTGGTGCGTCCAT-3′.

### 2.11. Bone Regeneration Analysis

After 3 and 12 weeks, the rats were sacrificed and a biopsy of the tibiae was performed. The samples were fixed in 4% paraformaldehyde for 24 h, followed by decalcification in 5% nitric acid for 3 days. After embedding the bone sections in paraffin blocks, histological sections were taken (5-µm thickness) using a microtome (Shandon™ Finessen 325, Thermo Electron Corporation). For histological analysis, all bone sections were stained with H&E. The indicators of bone regeneration were intraosseous acute inflammation (IAI), intraosseous chronic inflammation (ICI), periosteal inflammation (PI) and bone necrosis (BN), using the parameters described by Smeltzer et al. [22,23].

### 2.12. Statistical Analysis

For statistical analysis, the SAS software package (v9.2; SAS Institute, Cary, NC, USA) was used to perform one-way ANOVA. All data are expressed as means ± SD. A value of *p* < 0.05 was taken to indicate statistical significance.

## 3. Results

### 3.1. Cell Characterization by Immunocytochemistry

**Figure 1:**
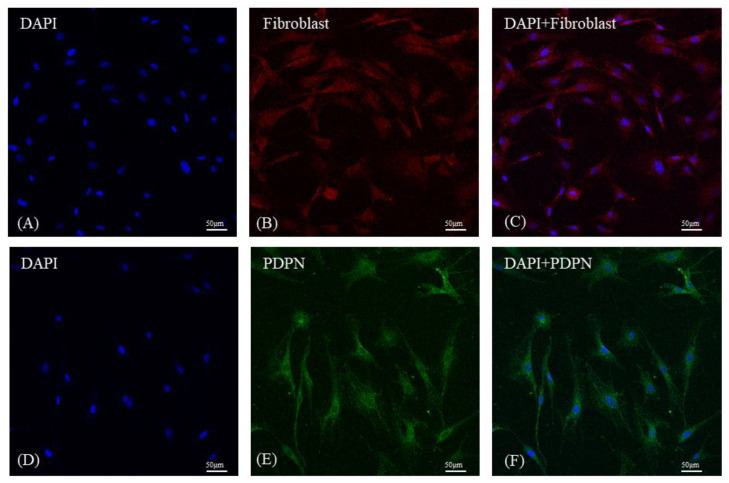
Characterization of isolated primary fibroblasts and osteocytes by immunocytochemistry. (**A**) The nucleus was stained with 4′,6-diamidino-2-phenylindole dihydrochloride (DAPI; magnification = 100×). (**B**) Goat anti-rabbit IgG H&E (Alexa Fluor®488; antibody against anti-fibroblast activation protein) was used to label fibroblasts; rat monoclonal IgG2a antibody was used to stain the cytoskeleton. (**C**) Merged ICC stain image showing the morphology and location of Fibroblast protein (Red) and cell nuclei (Blue). (**D**) The nucleus was stained with 4′,6-diamidino-2-phenylindole dihydrochloride (DAPI; magnification = 100×). (**E**) A FITC-conjugated anti-podoplanin antibody was used to label osteocytes, and the normal mouse IgG1 AlexaFluor®488 antibody was used to stain the cytoskeleton. Osteocytes nuclei were stained with DAPI (magnification = 100×). (**F**) Merged ICC stain image showing the morphology and location of Osteocyte protein (Green) and cell nuclei (Blue).

In cases of isolated primary fibroblasts, a goat anti-rabbit IgG H&L antibody was used to detect reactions between fibroblast activation protein and cytoskeletal proteins (green fluorescence), with nuclei stained blue using DAPI (Figure 1A). Similarly for osteocytes, the FITC-conjugated anti-podoplanin polyclonal antibody was used to stain cytoskeletal proteins (green area); nuclei were stained blue using DAPI (Figure 1B).

### 3.2. Cell Viability

**Figure 2:**
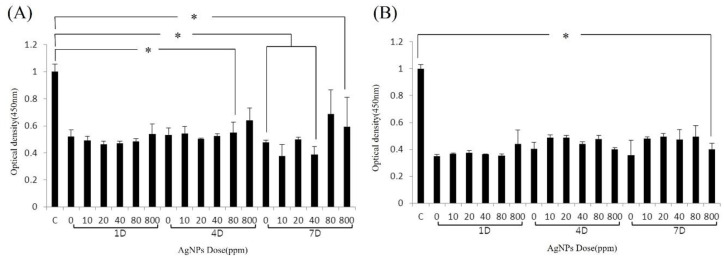
Effect of AgNP bone cement on cell viability. The viability of isolated primary fibroblasts (**A**) and osteocytes (**B**) treated with AgNP bone cement on days 1 (1D), 4 (4D) and 7 (7D) was determined by CCK-8 assay. * *p* < 0.05 (Mann-Whitney U test).

The bone cement (no Ag) was itself cytotoxic to primary fibroblasts and osteocytes. On days 1 (1D), 4 (4D) and 7 (7D) of culturing with bone cement, cell viability decreased to approximately 50–60% of non-cement-exposed cells (cell-only group; Figure 2A,B). The addition of 10, 20, 40, 80 and 800 ppm AgNP to the bone cement decreased cell viability compared to the bone cement-only groups (Figure 2A,B). Fibroblast viability at 1D was approximately 50–55% that of the cell-only group. There were no significant differences between the 10, 20, 40, 80 and 800 ppm silver content bone cements (Figure 2A). At 4D, cell viability was approximately 55–60% of that of the control group. Notably, bone cement containing higher concentrations of AgNP (40, 80, 800 ppm) resulted in higher cell viability relative to the bone-cement-only group (0 ppm) and low AgNP (10, 20 ppm) concentrations. At 1D, there were no significant differences among the various concentrations of AgNP in terms of cell viability or cytotoxicity; however, at 4D cell viability and cytotoxicity values differed significantly among 20, 40 and 800 ppm AgNP. At 7D, the use of 40 ppm AgNP in bone cement was associated with a significant decrease in cell viability compared to the bone-cement-only group (0 ppm; Figure 2A). The use of AgNP in bone cement decreased osteocyte viability. The average viability of osteocytes was approximately 35–40% that of the control group at 1D and increased to approximately 40–50% at 4D and 7D (Figure 2B). Similar to fibroblasts, there were no significant differences among the 10, 20, 40, 80 and 800 ppm AgNP-supplemented bone cements in terms of cell viability.

## 4. Osteomyelitis Characterization

**Figure 3:**
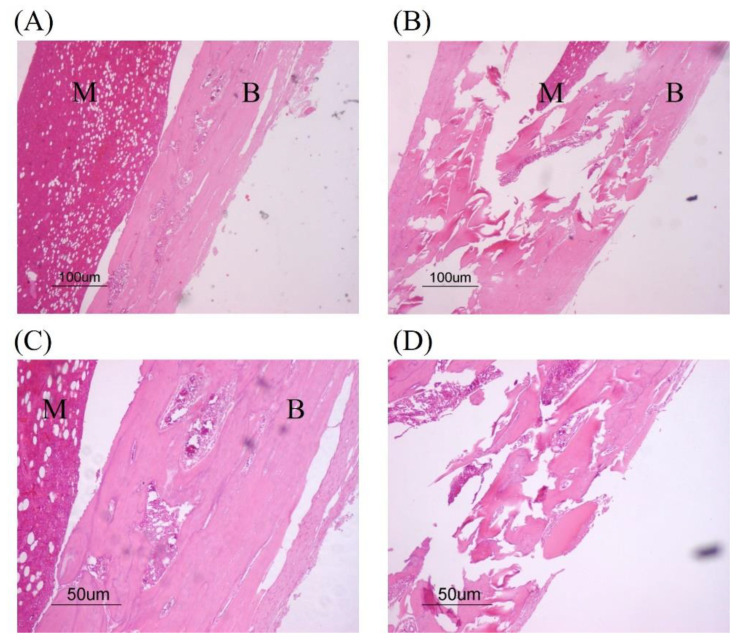
Characterization of osteomyelitis in infected bone. Four days after inducing osteomyelitis, the tibiae were removed and histological tissue sections created. Hematoxylin and eosin (H&E) staining was performed to assess the extent of osteomyelitis. (**A**,**C**): Osseous remodeling and tissue granulation; (**B**,**D**): bone destruction. Image magnification: (**A**,**B**), 40×; (**C**,**D**), 100×. B; Bone, M; Bone marrow.

Four weeks after MRSA injection, 5 of the 135 rats selected randomly were euthanized. The right tibiae of the rats were subjected to biopsy, to confirm the induction of osteomyelitis. Osseous remodeling, tissue granulation and bone destruction were analyzed by H&E staining (Figure 3).

## 5. Bacterial Culture of Infected and Treated Tibiae

**Figure 4:**
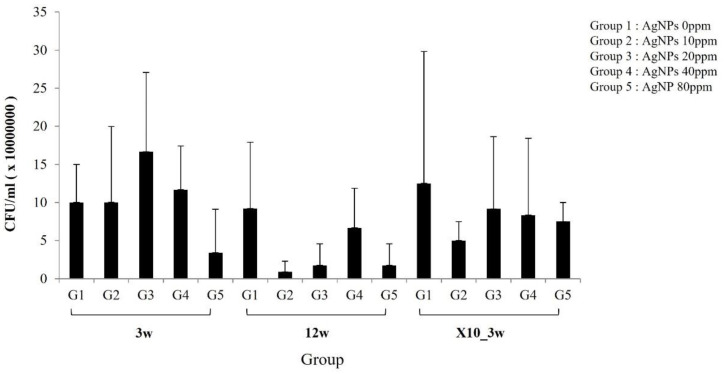
Bacteria culture. After 3 and 12 weeks of inserting AgNP-impregnated bone cement, the tibiae were removed and bacteria were cultured to determine the antibacterial effect. (Silver concentrations were as follows: G1; 0, G2; 10, G3; 20, G4; 40, G5; 80 PPM, where ×10 refers to a 10-fold increase in AgNP concentration).

The results of bacterial culture according to exposure duration and AgNP concentration (baseline and 10× dose) are shown in Figure 4. At week 3, there were no differences between the control and treatment groups in terms of bacterial colony counts, but there was a trend of decreasing colony counts in G5. At week 12, bacteria numbers were 10-fold lower in the treatment group compared to the control group, but no silver dose-dependency was observed (Figure 4).

## 6. Detection of MRSA Using RT-PCR

**Figure 5:**
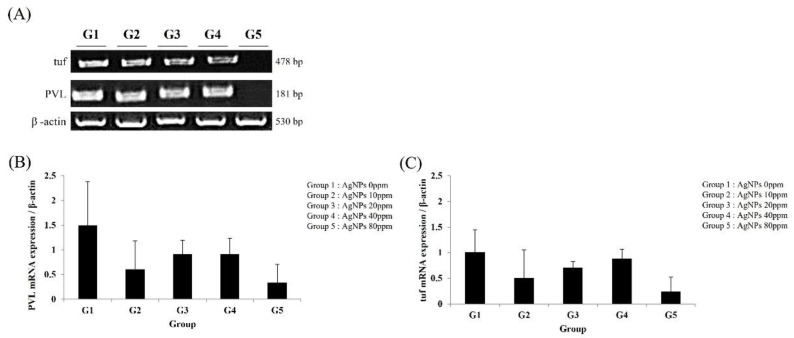
Analysis of *PVL* and tuf expression at 3 weeks using reverse transcription-polymerase chain reaction (RT-PCR). Three weeks after inserting the bone cement, the tibiae were removed, RNA was isolated and RT-PCR was performed using primers for *PVL* and tuf to determine the antibacterial effects of AgNPs. The loading control was β-actin. (**A**) Electrophoretic gel image showing the amplified gene transcripts; (**B**) quantification of *PVL* expression; (**C**) quantification of tuf expression. The AgNP concentrations used were as follows (ppm): G1, 0; G2, 10; G3, 20; G4, 40; G5, 80.

**Figure 6:**
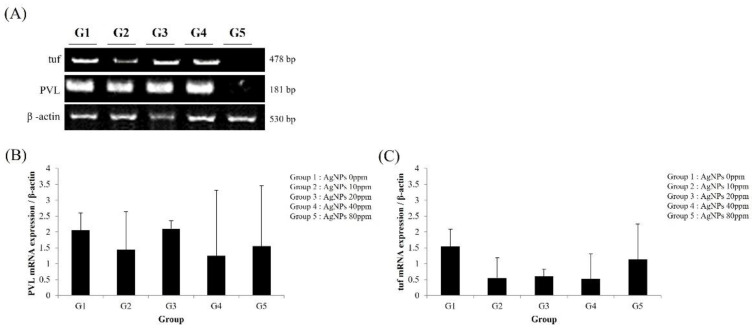
Analysis of *PVL* and tuf expression at 12 weeks by RT-PCR. Twelve weeks after inserting the bone cement, the tibiae were removed, RNA was isolated and RT-PCR was performed using primers for *PVL* and tuf to determine the antibacterial effects of AgNPs. The loading control was β-actin. (**A**) Electrophoretic gel image showing the amplified gene transcripts; (**B**) quantification of *PVL* expression; (**C**) quantification of tuf expression. The AgNP concentrations used were as follows (ppm): G1, 0; G2, 10; G3, 20; G4, 40; G5, 80.

**Figure 7:**
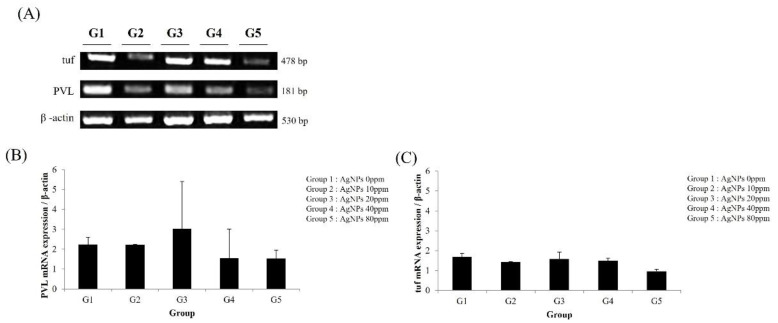
Analysis of *PVL* and tuf expression at 3 weeks using RT-PCR for the 10× AgNP concentration group. Three weeks after inserting the bone cement, the tibiae were removed, RNA was isolated and RT-PCR was performed using primers for *PVL* and tuf to determine the antibacterial effects of AgNPs. The loading control was β-actin. (**A**) Electrophoretic gel image showing the amplified gene transcripts; (**B**) quantification of *PVL* expression; (**C**) quantification of tuf expression. The AgNP concentrations used were as follows (ppm): G1, 0; G2, 10; G3, 20; G4, 40; G5, 80.

After 3 weeks of AgNP bone cement use, MRSA levels in infected tibiae were determined using the *PVL* and tuf genes. PLV gene expression was lower in groups treated with AgNP bone cement (G2–G5) compared to the bone-cement-only group (G1) (Figure 5A,B). Similarly, the AgNP bone cement-treated groups exhibited reduced tuf expression compared to the bone-cement-only group (Figure 5A–C). However, there were no significant differences according to AgNP dose. After 12 weeks of AgNP bone cement use, MRSA levels in infected tibiae were determined using the *PVL* and tuf genes. PLV gene expression was lower in groups treated with AgNP bone cement (G5) compared to the bone-cement-only group (G1) (Figure 6A,B). Similarly the AgNP bone cement-treated groups exhibited reduced tuf expression compared to the bone-cement-only group (Figure 6A–C). After 3 weeks of 10× AgNP bone cement use, MRSA levels in infected tibiae were determined using the *PVL* and tuf genes. PLV gene expression was lower in groups treated with AgNP bone cement (G5) compared to the bone-cement-only group (G1) (Figure 7A,B). Similarly, the AgNP bone cement-treated groups exhibited reduced tuf expression compared to the bone-cement-only group (Figure 7A–C).

## 7. Bone Regeneration

**Figure 8:**
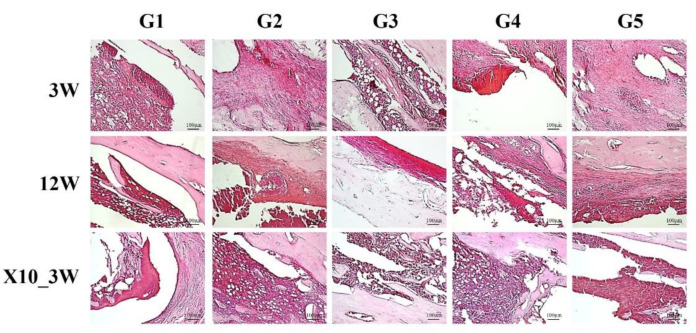
Assessment of bone regeneration. Three and twelve weeks after inserting the AgNP bone cement, three rats per group were euthanized. Tibiae biopsies were fixed using 4% paraformaldehyde and histological tissue sections were produced from paraffin blocks. H&E staining was used to assess the degree of bone regeneration. The AgNP concentrations used were as follows (ppm): G1, 0; G2, 10; G3, 20; G4, 40; G5, 80. Magnification = 100×.

**Figure 9:**
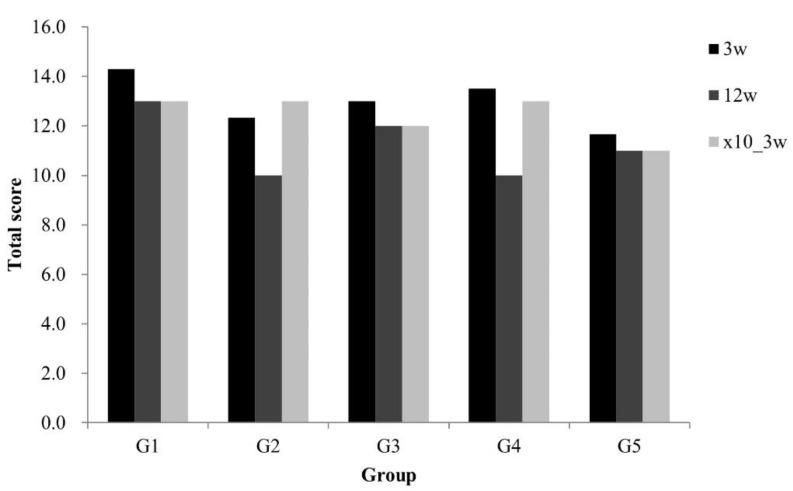
Quantification of bone regeneration. Using tibiae tissue sections (see Figure 8), the extent of intraosseous acute inflammation, intraosseous chronic inflammation, periosteal inflammation and bone necrosis was assessed on scales ranging from 0 to 4; total scores ranged from 0 to 16.

Concerning bone regeneration, the 3-week bone cement-only group (G1) exhibited severe IAI, ICI, PI and BN. Furthermore, a high degree of inflammation was observed in the 12-week bone cement-only (G1) group. However, the inflammation and necrosis levels of the AgNP bone cement-treated groups (G2–G5) were lower than those of the controls, indicating decreased infection and, possibly, improved bone regeneration. Furthermore, compared to the baseline AgNP concentration groups, the 10× concentration groups did not differ in terms of degree of inflammation or necrosis (Figure 8 and Figure 9).

## 8. Antimicrobial Activity of AgNPs against Resistant *MRSA*

**Figure 10:**
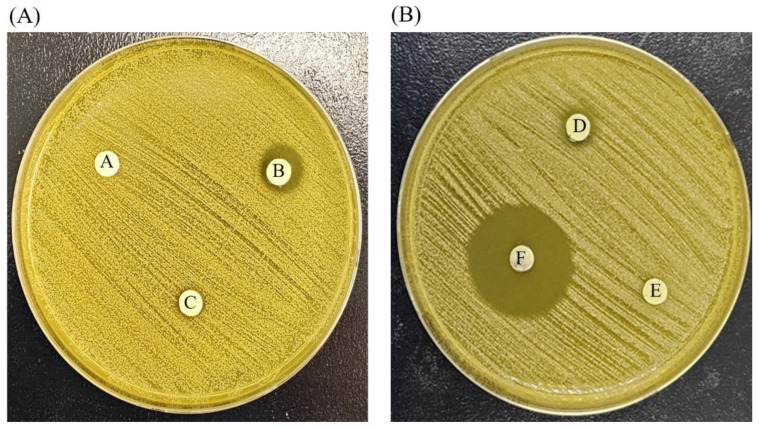
Disc diffusion test. The effect of silver nanoparticles on MRSA after incubation at 37 °C for 24 h on mannitol salt agar (KP) plate. (**A**) After incubating MRSA, the discs, which contained AgNP solutions diluted in PBS to concentrations of 0, 10, and 20ppm, were placed on the plate. (**B**)After incubating MRSA, the discs, which contained AgNP solutions diluted in PBS to concentrations of 40, 80, and 800ppm, were placed on the plate. A: 0 PPM, B: 10 PPM, C: 20 PPM, D: 40 PPM, E: 80 PPM, F: 800 PPM.

To confirm the antimicrobial activity of AgNPs against resistant MRSA, MRSA was cultured after treatment with AgNPs at various concentrations. The diameters of inhibition zones were measured in triplicate and ranged between 4.1 and 13.3 mm. The disks treated with 10, 40 and 80 ppm AgNPs showed inhibition zone diameters ranging from 3.5 to 4.8 mm. The diameter of the inhibition zone measured from the disk treated with 20 ppm AgNPs was 5.2 mm, while the disk treated with 800 ppm AgNPs exhibited a greater antimicrobial effect than the untreated disk (Figure 10).

## 9. Discussion

Osteomyelitis is difficult to treat because it involves bone and because the relapse rate is high [23]. Chronic osteomyelitis is also associated with high morbidity, although mortality is low [7,24]. Osteomyelitis is treated and managed surgically, in combination with antibiotic treatment (typically long-term; 6 to 12 weeks); such long-term treatment is a burden to both patients and healthcare systems [25]. This study examined the utility of AgNP bone cement for the treatment of osteomyelitis. Molecular techniques, such as gene sequence–based genotyping by polymerase chain reaction (PCR), became powerful tools in the diagnosis of bacterial species [26]. We investigated, by PCR determination, the presence of differentiate *Staphylococcus aureus*. In this study, Staphylococcus-specific PCR primers targeting the tuf and *PVL* genes were used. The tuf gene is evolutionary conserved and was shown to be more powerful and discriminative than the 16S rRNA gene in identifying and distinguishing Staphylococcus species [26]. The *PVL* gene is known to be one of the genes found in *Staphylococcus aureus* [27]. The PCR analysis using tuf and *PVL* genes allows for the detection of *Staphylococcus aureus*, and these results provide important information for infectious diseases. The bacterial culture results showed no significant difference in the bacterial counts between the control group and the treatment group. However, at 3 weeks and 12 weeks, the G5 group showed a tendency to decrease in bacterial counts compared to the G1 group. Similarly, PCR results also confirmed that at 3 weeks and 12 weeks, the G5 group showed a tendency to decrease in bacterial counts compared to the G1 group. Statistical significance could not be confirmed, as only three samples were analyzed. Nevertheless, these results demonstrate that silver nanoparticles-loaded bone cement can reduce bacterial counts against MRSA. At present, antibiotic-containing bone cement is among the most-popular osteomyelitis therapies; the antibiotics used must be specific to the bacterial infection being treated. AgNP exhibits broad antibacterial activity, as demonstrated by several studies. Silver, silver ions and AgNPs are used widely in the medical field. Sensitive wounds (burns or diabetic lesions) are treated using silver-containing wound dressings [28,29]. Silver-containing dressing materials confer particular benefits for infected diabetic wounds by facilitating re-epithelialization and granulation tissue proliferation, and by alleviating inflammation and reducing necrosis [30,31]. The antibacterial activity of AgNPs is mediated by two mechanisms: (i) interactions between AgNPs and the thiol group (-SH) of bacterial proteins; and (ii) ROS generation. Both of these mechanisms result in destruction of bacterial cell walls and membranes, interruption of DNA replication and protein synthesis and damage to DNA [32]. However, AgNPs also affect normal cells, in which increased apoptosis, high levels of inflammatory cytokines and DNA damage were reported [33]. Generally, the common treatment methods for bone infections include antibiotic administration, local nondegradable drug vehicles and aggressive surgical debridement [34,35]. However, the administration of antibiotics for bone infection treatment includes the problem of potentially forming biofilms [36]. Nanoparticles are known to be a useful tool in preventing biofilm formation due to their small size [37]. Silver nanoparticles (AgNPs) are well known potent antibacterial nano agents [38]. Additionally, AgNPs have an antibacterial effect against the Gram-positive bacterial pathogen *Staphylococcus aureus* [38,39,40]. In our in vivo study, the antibacterial effect of AgNP bone cement was evaluated by culturing bacteria from infected (and treated) tibiae. Infected bones treated with AgNP bone cement for 12 weeks had lower levels of bacteria (higher antibacterial activity) compared to those treated for 3 weeks. Therefore, similar to any other antibacterial treatment regimen, longer-duration AgNP bone cement treatment resulted in a decrease in bone infection. At 3 weeks, the antibacterial activity of the 10× AgNP dose was superior to those of the baseline dose and control (no AgNPs) groups, as indicated by more-rapid antibacterial activity. In addition to their antibacterial effects, AgNPs are also toxic to normal cells. The mechanism underlying this effect involves pinocytosis and endocytosis, during which AgNPs penetrate and accumulate in the cytoplasm of normal cells, inducing the formation of reactive oxygen species (ROS) by interacting with the products of mitochondrial cellular respiration, and eventually increasing intracellular oxidative stress. This results in increased inflammatory cytokine production and damage to DNA [41,42,43]. AgNPs exhibited limited cytotoxicity to osteocytes, although there was cytotoxicity to bone cement. Compared to the control (bone cement) group, the bone cement-only treatment group (no AgNPs) exhibited almost 50% lower cytotoxicity, indicating that the bone cement itself was cytotoxic. This could be overcome by using a different type of bone cement with reduced (or, ideally, completely absent), cytotoxicity to bone cells. Based on the bacterial culture results, the group treated with AgNP did not show statistically significant differences compared to the control group. However, at 3 weeks and 12 weeks, there was a tendency for the bacterial count to decrease in G5, which was treated with AgNP. In particular, at 12 weeks, the group treated with a higher (10×) dose of AgNP (G2–G5) showed a tendency for the bacterial count to decrease compared to the cement-treated group (G1), which was not treated with AgNP. Silver nanoparticles showed potential as an antimicrobial agent by inhibiting the growth of *Staphylococcus aureus* and penetrating the cell membrane and cell wall of bacterial cells, thereby destroying nucleic acids and proteins inside the cells [44]. To evaluate the antibacterial activity of silver nanoparticles (AgNPs) against Methicillin-resistant *Staphylococcus aureus* (MRSA), we measured the diameters of zones of growth inhibition using a disc diffusion assay and analyzed the results. Through a disk diffusion test, we confirmed that the growth of bacteria was inhibited on the disks treated with AgNP. These results indicate that there is a zone of inhibition of bacterial growth around the AgNP disk, suggesting that AgNP has antibacterial activity and can inhibit the growth of MRSA, which is one of the Gram-positive bacteria. Silver nanoparticles were shown to be effective in inhibiting the growth of *Staphylococcus aureus*, and their size, concentration and processing time were found to affect bacterial growth [45].

Silver nanoparticles have antimicrobial properties, making them highly versatile and useful not only in the medical field, but also in various other fields [46]. However, because the bone cement assessed in this study is widely used in the medical field, we aimed to determine the efficacy of AgNP incorporation in terms of treatment of osteomyelitis compared to traditional antibiotic regimens. At 3 and 12 weeks after the implantation of silver nanoparticles loaded-bone cement, the rats in each group were euthanized and the relevant bone tissues were harvested for RT-PCR. It is well known that studies evaluating bone formation often examine at 3 and 6 weeks or at 12 weeks. High-strength biodegradable zinc alloy implants with antibacterial and osteogenic properties for the treatment of MRSA-induced rat osteomyelitis [47,48]. As we used a large number of rats (total of 135) in vivo, there was a limitation for evaluating at 6 weeks in this study. In this way, our findings make an important contribution to the advancement of osteomyelitis treatment.

The antibacterial mechanism of chemical agents typically involves specific binding to the surface and metabolism of microorganisms, particularly in Gram-positive bacteria where they target the peptidoglycan layer [49], i.e., the Gram-positive bacteria including MRSA (methicillin resistant *Staphylococcus aureus* [50]). Our study is the first to evaluate the effect of silver nanoparticles-loaded bone cement for the treatment of MRSA-induced osteomyelitis. After using silver nanoparticles-loaded bone cement, bone tissue was extracted at 3 and 12 weeks, and the MRSA levels in the infected tibia were investigated using *PVL* and tuf genes. It was found that the expression of *PVL* and tuf genes showed a lower tendency in the AgNP-treated bone cement group (G2–G5) compared to the non-AgNP-treated bone cement group (G1), although there was no statistically significant difference. In the histological results, it was observed that the inflammation and necrosis levels in the AgNP bone cement-treated group (G2–G5) decreased compared to the non-AgNP-treated bone cement group (G1). In this paper, we described the potential impact and therapeutic efficacy of silver nanoparticles loaded-bone cement on MRSA induced-osteomyelitis.

However, further research is required to identify the optimal bone cement composition and AgNP concentration, particularly for clinical use. Our findings demonstrated the antibacterial effect of AgNP bone cement; we are planning further studies to determine the optimal AgNP dose and treatment duration to achieve the best outcome in the treatment of osteomyelitis. This study confirmed the antibacterial effect of AgNP bone cement; we believe that AgNP bone cement represents an effective option for the long-term treatment of osteomyelitis.

## 10. Conclusions

This study indicates that AgNP-impregnated TTCP-DCPD bone cement could be considered to treat osteomyelitis.

## Data Availability

Not applicable.

## Abbreviations

AgNP = silver nanoparticle, MRSA = Methicillin-resistant *Staphylococcus aureus*, TTCP-DCPD = tetracalcium phosphate-dicalcium phosphate dihydrate, RT-PCR = Reverse transcription polymerase chain reaction.
